# New distribution patterns of *Dirofilaria immitis* in Italy

**DOI:** 10.3389/fvets.2023.1162403

**Published:** 2023-05-04

**Authors:** Ettore Napoli, Giovanni De Benedetto, Lavinia Ciuca, Antonio Bosco, Riccardo Paolo Lia, Vincenzo Veneziano, Marcos Antônio Bezerra Santos, Domenico Otranto, Laura Rinaldi, Emanuele Brianti

**Affiliations:** ^1^Department of Veterinary Sciences, University of Messina, Messina, Italy; ^2^Department of Veterinary Medicine and Animal Production, University of Napoli, Napoli, Italy; ^3^Department of Veterinary Medicine, University of Bari, Bari, Italy; ^4^Faculty of Veterinary Sciences, Bu-Ali Sina University, Hamedan, Iran

**Keywords:** heartworm disease, *Dirofilaria repens*, *Acanthocheilonema reconditum*, epidemiology, canine filariosis, Italy

## Abstract

In recent decades, the number of autochthonous cases and foci of *Dirofilaria immitis* in dogs from southern regions has increased considerably, suggesting that the distribution of the species is not limited to northern Italian regions. This epidemiological picture emerges from case reports or studies in specific locations where outbreaks of heartworm disease have occasionally been reported together with the presence of mosquito vectors. To obtain a more comprehensive picture of the current distribution of *D*. *immitis* in southern Italy, a multicenter cross-sectional survey of canine filariasis was conducted. Owned and sheltered dogs (*n* = 1,987) were included in the survey regardless their breed, attitude and/or sex. All included dogs were older than 1 year and had no history of chemoprophylactic treatment against filarioses. A blood sample was collected from enrolled dogs and screened by modified Knott’s test and, when positive, tested using *D*. *immitis* specific ELISA rapid test (SNAP 4DX, IDEXX). The overall microfilaremia prevalence was 17% (*n* = 338) being single-species infection (92.6%) more common that mixed (7.4%). Remarkably, *D*. *immitis* was the most frequent species detected with an overall prevalence of 11.4% (*n* = 227), followed by *Dirofilaria repens* (*n* = 74; 3.7%), and *Acanthocheilonema reconditum* (*n* = 12; 0.6%). Sheltered dogs were significantly more infected by *D*. *immitis*, as well as mongrel dogs and animals housed in rural areas. Data here reported indicate that *D*. *immitis* is largely present in southern Italy, raising awareness about the necessity of proper screening and chemoprophylactic treatments in exposed animals.

## 1. Introduction

Canine filariosis caused by *Dirofilaria repens* and *Acanthocheilonema reconditum* have been constantly reported as endemic in southern Italian regions, while *Dirofilaria immitis* is considered sporadic in the area, and negligible its risk of transmission to dogs (1–4).

*Dirofilaria immitis* and *Dirofilaria repens* (Spirurida, Onchocercidae) are filarioids of major veterinary and medical concern, because of their zoonotic potential (5–7). While *D*. *immitis* is the causative agent of canine cardiopulmonary heartworm disease (HWD) and causes a serious disease with a chronic evolution, *D*. *repens* causes subcutaneous filariosis, and is of minor veterinary relevance (7, 8). Both diseases are transmitted by mosquitoes, being their epidemiology linked with the presence of proper vector species and of suitable reservoirs (9). To date, more than 70 mosquito species belonging to the genera *Aedes* (*Ochlerotatus*), *Anopheles* and *Culex* have been recognized as competent or putative vectors of both filaria species, with some molecular evidence of *D*. *immitis* in biting-midges (10). In addition, *Acanthocheilonema reconditum* is a worldwide distributed flea-transmitted filarioid, which is considered as the most prevalent filaroid infesting dogs in the Mediterranean Basin (11–13).

The distribution patterns of *Dirofilaria* spp. may be influenced by several factors, including the increase in vector population, the introduction of invasive species (e.g., *Aedes albopictus* and *Aedes koreicus*), the global movements of pets (2, 14), as well as the non-adoption of chemoprophylaxis in endemic regions (15). Therefore, significant changes in the epidemiology of the genus have been observed in the last decades (3, 16).

*Dirofilaria repens* has increased its prevalence in areas where it has already been reported and its distribution range has expanded into new areas of Europe, with an increase of clinical cases in both dogs and humans (2, 8). Autochthonous *D*. *repens* infections have been found in dogs in most European countries, from Portugal to Russia (2). Although the *D*. *repens* was considered endemic in Mediterranean countries (Italy, Southern France, and Greece), the increase of its prevalence has been recently reported in north-eastern and eastern Europe (2). Similarly, a progressive expansion of *D*. *immitis* to southern Italian regions has been observed in the last years (17, 18), and foci featured by high prevalence rete reported in area previously thought as non-endemic [i.e., Apulia region, (19); Linosa island, Sicily, (15)].

Given the paucity of scientific information about the distribution of canine filarioses in southern Italian regions, a multicenter cross-sectional survey was conducted to obtain a more comprehensive picture of their current presence and distribution.

This study aimed to conduct a cross-sectional multicentric survey, to investigate the occurrence of canine filariosis in southern Italy by means of a large multicentre epidemiological study.

## 2. Materials and methods

### 2.1. Ethical statement

The study was conducted according to CVMP/VICH/595/1998—VICH GL 9: Good Clinical Practice and it was approved by the Ethical Committee of the Department of Veterinary Sciences of the University of Messina (no. 059/2021).

### 2.2. Study population and sampling

The survey was conducted according to CVMP/VICH/595/1998—VICH GL 9: Good Clinical Practice and signed owner informed consent (OIC) was obtained for all the dogs included in the study.

The study was conducted in Lazio, Campania, Apulia, Basilicata, Calabria and Sicily and it was coordinated by three Regional Units (RUs): RU Campania; RU Puglia and Basilica, and RU Sicily, Calabria, and Lazio. From July to December 2021 owned and sheltered dogs, older than 12 months, in good general health condition and not under any filaricidal and/or microfilaricidal treatments were enrolled in the study. However, in few cases, animals younger than 12 months were included in the study as they shared the same pen/house with other enrolled dogs. The minimum sample size for each RU was 383 dogs assuming a confidence of 95, 5% of margin of error, unlimited population size (> 10,000), and setting the expected prevalence at 50%. Therefore, the expected sample size was 1,149 dogs. The RUs were supported by local veterinary facilities (i.e., clinics and veterinary hospitals) for the selection of dogs to be included in the survey as well as for the first screening analyses (i.e., Knott’s test). Briefly each dog, considered eligible for the inclusion in the study, underwent a complete physical examination, and a blood sample was collected from a peripheral vein (e.g., jugular or cephalic). Two blood aliquots of 1 mL were collected into an anticoagulant tube (K_3_EDTA) and used for Knott’s test and about 3 mL of blood were collected in tube with cloth activator, centrifuged (1.678 *g* × 10 min) and the serum stored and sent to the RU of reference for ELISA rapid antigen test. When samples scored positive for the presence of microfilariae at the Knott’s test the stored aliquots of blood and serum were sent to the RU of reference for further confirmation and identification of microfilariae.

### 2.3. Laboratory procedures

Blood samples were examined by modified Knott’s test (20). The microfilariae eventually detected were identified at species level using morphometric criteria (21), counted and the load expressed as microfilariae per mL of blood (mfs/mL). Samples positive to modified Knott’s test were analyzed by ELISA rapid test (SNAP 4DX, IDEXX laboratories, Westbrook, ME, United States) specific for antibodies against *Anaplasma* spp., *Borrelia* spp., *Ehrlichia* spp., and antigen of *D*. *immitis* following the manufacturer recommendations.

### 2.4. Statistical analyses

Descriptive statistics was used to analyze the data. For each filarial species, the epidemiological indices of infection were calculated according to Bush et al. (22), and Pearson’s chi-square analysis was applied to evaluate differences of filarial species and dogs’ variables (i.e., geographical origin; status, owned/sheltered; gender; age; breed; pure-breed/mongrel; type of housing; and habitat). All analyses were performed using GraphPad Prism version 8.1 for MacOx (GraphPad Software, San Diego California United States, www.graphpad.com). A value of *p* of 0.01 was used as a threshold to assess significant differences among values. Sample size was calculated with an on-line sample size calculator (Calculator.net; https://www.calculator.net/sample-size-calculator.html).

## 3. Results

A total of 1,987 dogs were enrolled in the survey (Table 1). The study population was balanced for sex and typology (i.e., shelter/kennel or private owned) while most of the dogs were living in rural or suburban areas and maintained permanently outdoor or with a constant outdoor (Table 1).

**Table 1:** Description of the study population enrolled in the epidemiological survey of filarial worm distribution in Italy.

		Status		Sex		Breed		Attitude			Lifestyle			Areal		
Region	*N*.	Shelter	Owned	Male	Female	Mix breed	Pure breed	Pets	Guard	Hunting	Indoor	Outdoor	Mix	Rural	Sub-urban	Urban
Sicily	607	223	384	289	318	434	173	444	31	132	35	420	152	234	323	50
Calabria	174	68	106	90	84	104	70	144	9	21	15	64	95	91	44	39
Basilicata	41	0	41	24	17	18	23	41	0	0	12	21	8	18	11	12
Puglia	417	385	32	222	195	395	22	417	0	0	0	0	417	417	0	0
Campania	598	258	340	322	176	216	382	251	46	301	9	546	43	471	100	27
Lazio	150	40	110	74	76	101	49	133	8	9	44	60	46	32	65	53
Total	1,987	974	1,013	1,021	866	1,268	719	143	94	463	115	1,111	761	1,263	543	181

In Sicily, Calabria, and Campania regions, the number of dogs from shelter or owned was balanced, while those enrolled in Apulia were mainly from shelters. In Lazio and Basilicata, the number of owned dogs exceeded those from shelters. Apart from Basilicata, in all other regions, the number of mongrel dogs was larger than pure-breed dogs (Table 1). The mean age was 70.7 months (±13.3; min 7.5—max 201.7 months). The study population was grouped into six age classes: <12 months, 12–36, 37–72, 73–120, 121–180, and > 180 months. The study population was almost equally distributed among the six age-classes while only 1.63 and 0.65% of the enrolled dogs were < 12 or > 180 months, respectively.

According to the collected history of dogs and the clinical examination performed by a member of the RU staff or by the veterinary practitioners that included the animals no clinical signs of filarial infection (e.g., cough, dyspnoea for HWD or presence of nodules or other skin disorders for *D*. *repens* and *A*. *reconditum* infections) were observed.

Overall, 338 out of the 1,987 enrolled dogs (i.e., 17.01%) were positive to circulating microfilariae, being monospecific infection (i.e., 313, 92.60%) more common that mixed ones (i.e., 25, 7.40%; Table 2). *Dirofilaria immitis* was the most frequently diagnosed species with an overall prevalence of 12.63% (i.e., 251, 74.26% of positive cases), followed by *D*. *repens* (i.e., 98, 29.00% of positive cases) and *A*. *reconditum* (i.e., 14, 4.14% of positive cases) with prevalence of 4.93 and 0.70%, respectively. The commonest mixed infection was that caused by *D*. *immitis* and *D*. *repens*. In all the investigated regions, *D*. *immitis* was the commonest filarial species but not in Campania, while *A*. *reconditum* was retrieved exclusively in Sicily and Calabria regions. Basilicata was the sole region in which any filarial infection was detected, but it was also the region with the lowest number of included dogs (i.e., 41).

**Table 2:** Number of dogs tested positive to Knott’s test for the detection of filarial infection in the six-region investigated in the study and number of dogs positive to the single species and for mixed infections.

Region	*N*. dogs	Positive (%)	*Dirofilaria immitis*	*Dirofilaria repens*	*Acanthocheilonema reconditum*	Mixed (%)	Di + Ar	Di + Dr	Dr + Ar
Sicily	607	103 (16.97)	52 (8.57)	40 (6.59)	10 (1.65)	1 (0.16)	0	0	1
Calabria	174	9 (5.17)	4 (2.30)	2 (1.15)	2 (1.15)	1 (0.57)	1	0	0
Basilicata	41	0 (−)	-	-	-	-	-	-	-
Apulia	417	195 (46.76)	163 (39.09)	21 (5.04)	0 (−)	11 (2.64)	0	11	0
Campania	598	27 (4.52)	4 (0.67)	11 (1.84)	0 (−)	12 (2.01)		12	
Lazio	150	4 (2.67)	4 (2.67)	-	-	-	-	-	-
Total	1987	338 (11.42)	227 (11.42)	74 (3.72)	12 (0.60)	25 (1.26)	1	23	1

(Di + Ar = mixed infection *Dirofilaria immitis* and *Acanthocheilonema reconditum*; Di + Dr. = mixed infection *Dirofilaria immitis* and *Dirofilaria repens*; and Dr. + Ar = mixed infection *Dirofilaria repens* and *Acanthocheilonema reconditum*).

In all the other regions, the prevalence of *D*. *immitis* was significantly higher compared to the other filarial species, being the higher observed in Apulia (i.e., 39.09%), followed by Sicily (i.e., 8.57%), Lazio and Campania (Table 2).

Most of the dogs enrolled in Apulia region were housed in shelter; indeed, sheltered dogs were more significantly infected (χ^2^ = 108.3755. *p* < 0.00001) compared to private owned dogs. This finding was valid for *D*. *immitis* (χ^2^ = 163.3427, *p* < 0.00001) but not for *D*. *repens* (χ^2^ = 5.8451, *p* = 0.01562) and *A*. *reconditum*, being these two latter species mainly found in owned dogs (χ^2^ = 5.1332, *p* = 0.023472).

Filarial infection was more common in mongrel dogs. No statistical difference was observed among sex (χ^2^ = 3.2075, *p =* 0.073304). Type of housing was a relevant variable, indeed, dogs kept mainly in indoor were less exposed to *D*. *immitis* infection, while no statistical differences were observed for *D*. *repens* and *A*. *reconditum*. Dogs housed in rural area were more exposed to filarial species transmitted by mosquitos, while no difference in the infection rate was observed between dogs living in suburban or urban areas. The results of statistical analyses are summarized in Table 3.

**Table 3:** Statistically significant differences observed between the filarial infection, the different filarial species, and the considered variables.

Species	Significant variable	χ^2^	*p* value	Significant
Total	Mongrel dogs	91.3596	< 0.00001	Yes
*Dirofilaria immitis*		92.7365	< 0.00001	Yes
*Dirofilaria repens*		13.3932	0.000253	Yes
*Acanthocheilonema reconditum*		1.5504	0.213076	No
Total	Age class (73–120)	122.5432	< 0.00001	Yes
*Dirofilaria immitis*		116.9510	< 0.00001	Yes
*Dirofilaria repens*		13.9711	0.002945	Yes
*Acanthocheilonema reconditum*		1.7685	0.41302	No
Total	Indoor housing	21.8372	0.00018	Yes
*Dirofilaria immitis*		25.444	< 0.00001	Yes
*Dirofilaria repens*		3.0748	0.214943	No
*Acanthocheilonema reconditum*		-	-	-
Total	Rural area	106.1568	< 0.00001	Yes
*Dirofilaria immitis*		80.7429	< 0.00001	Yes
*Dirofilaria repens*		7.8721	< 0.00001	Yes
*Acanthocheilonema reconditum*		2.4161	0.298772	No

## 4. Discussion

The high prevalence (12.63%) of *D*. *immitis* infection in dogs in southern Italy indicates that this mosquito-borne nematode is widespread also in the investigated geographical area and, therefore, should not be considered a “northern Italy parasite” anymore. Also, it is likely that the *D*. *immitis* prevalence herein reported is slightly underestimated as the screening diagnosis was performed only by modified Knott’s test, while heartworm antigen test was used only as confirmatory test (23). However, the prevalence observed in southern Italian regions is higher if compared to those of other some European countries (23, 24). The data herein reported corroborate what observed in a survey conducted on a large dataset in which the cumulative prevalence of *D*. *immitis* progressively increased in central and southern Italy regions in the last decade (17). Worthy of note, a similar picture has recently been observed in other Mediterranean countries such as Spain (25). On the other hand, *D*. *repens* infection rate seems to be steady as the prevalence here reported overlaps what previously described for southern Italy (2, 4). Conversely, *A*. *reconditum* presence decreased significantly compared to other surveys conducted in the same area (4, 11, 13). The increased prevalence of *D*. *immitis* is likely the result of a mix of variables such as the greater presence/abundance of competent mosquito vectors, the changing in the environment and climate, and the increased movement of dogs from and to endemic areas for trade and tourisms (17, 19). Therefore, the southwards spreading of *D*. *immitis* is not surprising, considering the climate and the average temperatures of these regions that are largely above the threshold (14°C) indicated for development of *Dirofilaria* spp. larvae to the infectious stage (26, 27) as well as for the development of several Culicidae (namely, *Ae*. *caspius*, *Ae*. *sticticus*, *Ae*. *vexans*, *Cx*. *modestus*, and *Cx*. *pipiens*) recognized as suitable *Dirofilaria* spp. vectors (28). Finally, the absence of systematic chemoprophylaxis treatments against filarioses in dogs living in southern regions is a major driver for the spread of the infections. In fact, the high prevalence rate in hyperendemic areas (e.g., 58.9% in Spain, (29); 22–80% in northern Italy, (6)) decreased after the regular use of preventative treatments (3, 24). On the other hand, the decreased prevalence of *A*. *reconditum* should be linked to the increased use of strategies effective for the control of ectoparasite (fleas and lice) infections (30, 31). In fact, the prevention of *Dirofilaria* spp. is mainly related to the use of chemoprophylaxis and only in less extent to blocking transmission from mosquitoes to dogs using repellents/insecticides (32), while the prevention of *A*. *reconditum* is exclusively related to the control of flea infestation.

In this study, most of the dogs infected by *Dirofilaria* spp. were hosted in shelter located in rural and suburban areas; often these dogs do not receive preventative treatment and have no protection against mosquito bites. The high prevalence of *D*. *immitis* infection in sheltered dogs indicates that dog communities represent a potential risk for amplification and spread of diseases. Therefore, it is of great importance to screen and correctly manage (i.e., treatment of infected animals and use preventive strategy) these animals for the presence of filarial infections. Infected sheltered dogs, are, indeed, regarded as an important parasite source for the mosquito vectors, being also a hazard for public health (19, 33, 34).

In conclusion, based on the above data, *D*. *immitis* should no longer be considered a sporadic parasite in southern Mediterranean regions but rather an endemic parasite. In the same manner, the prevalence rate of *D*. *repens* suggests that this species is still present and widely circulates in southern Italy. On the other hand, the widespread use of ectoparasiticide compounds led to a progressive reduction of *A*. *reconditum* infection rate.

Due to the zoonotic potential of both *D*. *immitis* and *D*. *repens* combined with the veterinary relevance of the former species (35), regular screening and strategic chemoprophylaxis treatments against infection are needed to minimize the risk of infection and limit the spread in southern Italian regions. Moreover, it is crucial for dog shelters to overcome the main challenges concerning the risks and management of HWD and other zoonotic vector-borne diseases by improving their protocols for diagnosis and prevention (e.g., systematic use of chemoprophylaxis and repellent products against vectors bites).

On light of this last statement is clear how veterinarians play a significant role in the prevention and should be more aware of their responsibility in controlling vector-borne zoonotic diseases. Veterinarians must increase their awareness not only on the new epidemiologic scenario of canine filarioses, but also they should be aware on the presence of specific guidelines for the diagnosis and management of *D*. *immitis* such as those released by the European Scientific Counsel Companion Animal Parasites (ESCCAP), the American Heartworm Society (AHS), and the European Society of Dirofilariosis and Angiostrongylosis (ESDA).

## Data availability statement

The raw data supporting the conclusions of this article will be made available by the authors, without undue reservation.

## Ethics statement

The animal study was reviewed and approved by Ethical Committee of the Department of Veterinary Sciences of the University of Messina. Written informed consent was obtained from the owners for the participation of their animals in this study.

## Author contributions

EB, DO, VV, and LR: conceptualization, resources, data curation, and supervision. EN, LC, AB, GB, and RL: methodology and investigation. EN, LC, GB, and LR: validation. EN: formal analysis. EN and EB: writing—original draft preparation. EB, EN, DO, VV, and LR: writing—review and editing. MB, LC, AB, GB, and RL: visualization. All authors contributed to the article and approved the submitted version.

## Funding

The authors declare that this study received funding from Boehringer Ingelheim. The funder was not involved in the study design, collection, analysis, interpretation of data, the writing of this article, or the decision to submit it for publication. The contribution by LR and DO was partially supported by EU funding within the NextGenerationEU-MUR PNRR Extended Partnership initiative on Emerging Infectious Diseases (Project no. PE00000007, INF-ACT).

## Conflict of interest

The authors declare that the research was conducted in the absence of any commercial or financial relationships that could be construed as a potential conflict of interest.

## Publisher’s note

All claims expressed in this article are solely those of the authors and do not necessarily represent those of their affiliated organizations, or those of the publisher, the editors and the reviewers. Any product that may be evaluated in this article, or claim that may be made by its manufacturer, is not guaranteed or endorsed by the publisher.
